# Psychiatric comorbidity profiles among suicidal attempters: A cohort study

**DOI:** 10.1192/j.eurpsy.2023.705

**Published:** 2023-07-19

**Authors:** Y. Sanchez-Carro, M. Diaz-Marsa, V. Fernandez-Rodrigues, W. Ayad-Ahmed, A. Pemau, I. Perez-Diaz, A. Galvez-Merlin, P. de la Higuera-Gonzalez, V. Perez-Sola, P. Saiz, I. Grande, A. Cebria, J. Andreo-Jover, P. Lopez-Peña, M. Ruiz-Veguilla, A. de la Torre-Luque

**Affiliations:** ^1^ Universidad Autonoma de Madrid; ^2^ Biomedical Research Networking Consortium for Mental Health (CIBERSAM ISCIII); ^3^ CIBERSAM ISCIII; ^4^ Universidad Complutense de Madrid; ^5^ Health Research Institute, Hospital Clínico San Carlos (IdISSC); ^6^Department of Personality, Assessment and Clinical Psychology, Faculty of Psychology; ^7^Department of Legal Medicine, Psychiatry and Pathology, Faculty of Medicine, Universidad Complutense de Madrid; ^8^ Health Research Institute, Hospital Clínico San Carlos (IdISSC); ^9^ Institut Hospital del Mar d’Investigacions Mediques (IMIM); ^10^Biomedical Research Networking Consortium for Mental Health (CIBERSAM ISCIII), Madrid, Spain; ^11^Universidad de Oviedo, Oviedo; ^12^ Bipolar and Depressive Disorders Unit, Hospital Clinic; ^13^Institute of Neurosciences, University of Barcelona; ^14^ August Pi i Sunyer Biomedical Research Institute (IDIBAPS); ^15^Biomedical Research Networking Consortium for Mental Health (CIBERSAM ISCIII), Barcelona; ^16^ Biomedical Research Networking Consortium for Mental Health (CIBERSAM ISCIII); ^17^Corporacio Sanitaria Parc Tauli, Sabadell; ^18^Instituto de Investigación del Hospital Universitario La Paz (IdiPAZ), Madrid; ^19^ Hospital Universitario Virgen del Rocio; ^20^Biomedical Research Networking Consortium for Mental Health (CIBERSAM ISCIII), Sevilla; ^21^Biomedical Research Networking Consortium for Mental Health (CIBERSAM), Madrid, Spain

## Abstract

**Introduction:**

More than 700,000 people die by suicide in 2019 globally (World Health Organitation 2021). Mental health problems constitute a risk factor for suicidal behavior and death by suicide (Hoertel et al. Mol Psychiatry 2015; 20 718–726). Different mental disorders have been related to different forms of suicidal ideation and behavior (Conejero et al. Curr Psychiatry Rep 2018; 20, 33) (Quevedo et al. Compr Psychiatry 2020; 102 152194). However, little is known on comorbidity profiles among suicide attempters.

**Objectives:**

The aim of our work was to identify the psychiatric comorbidity profiles of individuals who were admitted a hospital emergency department due to a suicide attempt. Moreover, it intended to know their clinical characteristics according to comorbidity profile.

**Methods:**

A sample of 683 attempters (71.30% female; M age= 40.85, SD= 15.48) from the SURVIVE study was used. Patients were assessed within the 15 days after emergency department admission. Sociodemographic (i.e., sex, age, marital status and employment status) and clinical data were collected. The International Neuropsychiatric Interview (MINI) was used to assess DSM-V Axis 1 mental health diagnoses and the Columbia Suicide Rating Scale (C-SSRS) to assess suicidal ideation and behavior. The Acquired Capacity for Suicide-Fear of Death Scale (ACSS-FAD), the Patient Health Questionnaire (PHQ-9) to assess the frequency of depressive symptoms during the past 2 weeks, and the General Anxiety Disorder-7 (GAD-7) scale to assess symptoms of worry and anxiety were also conducted. For the identification of comorbidity profiles, latent class analysis framework was followed considering diagnosis to each individual disorder as clustering variables. On the other hand, binary logistic regression was used to study the relationship between comorbidity profile membership and clinical factors.

**Results:**

Two classes were found (Class I= mild symptomatology class, mainly featured by emotional disorder endorsement; and Class II= high comorbidity class, featured by a wide amount of endorsed diagnoses) (see figure 1). Individuals from the High comorbidity class were more likely to be female (OR= 0.98, p<.05), younger in age (OR= 0.52, p< .01), with more depressive symptoms (OR=1.09, p<.001) and have greater impulsivity (OR= 1.01, p<.05).

**Image:**

**Figure:**
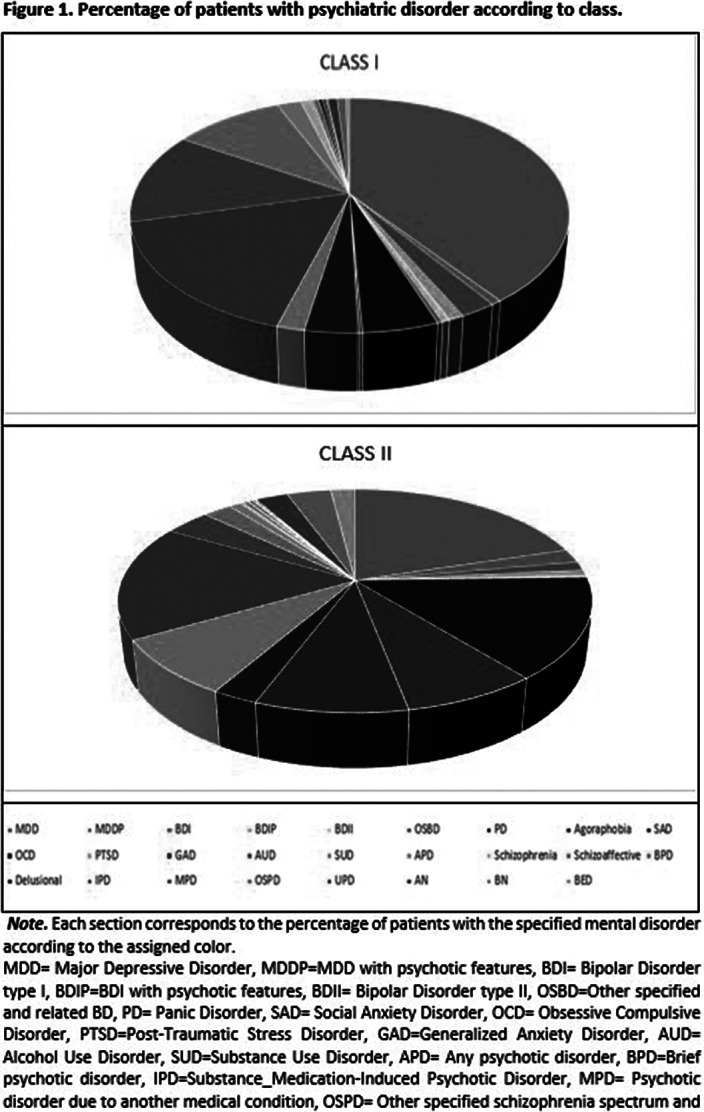

**Conclusions:**

We found two profiles of people with suicidal behavior based on the presence of mental disorders. Each of the suicidal subtypes had different associated risk factors. They also had a different profile of suicidal behavior.

**Disclosure of Interest:**

None Declared

